# First-morning urinary luteinizing hormone and bone age decomposition for diagnosis of central precocious puberty in girls: a two-step prediction model study

**DOI:** 10.3389/fendo.2026.1948363

**Published:** 2026-10-07

**Authors:** Miao Huang, Xiyan Lu, Peipei Liu, Guoqing Dong, Mingzhu Li, Wujin Wang, Jianxu Li

**Affiliations:** Shenzhen Maternity and Child Healthcare Hospital, Women’s and Children’s Medical Center, Southern Medical University, Shenzhen, Guangdong, China

**Keywords:** bone age, central precocious puberty, diagnostic accuracy, gonadotropin-releasing hormone stimulation test, prediction model, reference interval, urinary luteinizing hormone

## Abstract

**Objective:**

The diagnosis of central precocious puberty (CPP) in girls relies on the invasive gonadotropin-releasing hormone (GnRH) stimulation test. Urinary luteinizing hormone (LH) is a noninvasive candidate biomarker, but evidence from Chinese populations and its integration into multivariable prediction frameworks remain limited. We evaluated the diagnostic accuracy of first-morning urinary LH for CPP, established sex- and pubertal stage–specific reference intervals, and developed a two-step prediction model based on a bone age decomposition.

**Methods:**

In this single-center retrospective diagnostic accuracy study, we enrolled 808 children: 295 girls with early breast development (146 CPP, 149 non-CPP), 271 healthy girls, and 242 healthy boys (reference intervals only). First-morning urine was assayed for LH, follicle-stimulating hormone (FSH), and creatinine. Diagnostic performance was evaluated primarily in the 295 symptomatic girls, with an extended analysis in 566 girls including healthy controls; multivariable logistic regression decomposed bone age into chronological age (CA) and the BA–CA difference as separate predictors.

**Results:**

Urinary LH showed moderate accuracy for CPP in the primary symptomatic cohort (AUC 0.692, 95% CI 0.630–0.752), peaking in girls aged 7–8 years (AUC 0.708, 95% CI 0.621–0.785); the extended cohort gave similar results (AUC 0.712; secondary, spectrum-extended estimate). The LH-to-creatinine ratio offered no improvement. Basal serum LH was the superior single marker (AUC 0.868 vs 0.692, P < 0.001). The noninvasive Model 1 (CA + BA–CA + urinary LH + breast Tanner stage + uterine length) achieved a C-statistic of 0.853 (sensitivity 73.3%, specificity 83.2%), with CA, BA–CA, and urinary LH as independent predictors. Adding basal serum LH and FSH (Model 2) improved the C-statistic to 0.909; urinary LH retained independent significance (aOR 1.280, P = 0.024), whereas basal FSH was inversely associated with CPP. Bootstrap-corrected C-statistics were 0.838 (Model 1) and 0.893 (Model 2). Pubertal-onset cutoffs were >1.02 mIU/mL in girls and >0.95 mIU/mL in boys.

**Conclusions:**

Decomposing bone age into CA and BA–CA enables tiered CPP risk stratification: the noninvasive model supports initial screening without blood sampling, and the complete model refines risk once serum gonadotropins become available. Urinary LH is a clinically useful component of tiered diagnostic algorithms for CPP.

## Introduction

Central precocious puberty (CPP) results from premature reactivation of the hypothalamic–pituitary–gonadal (HPG) axis before 8 years of age in girls, leading to accelerated skeletal maturation, compromised adult height, and psychosocial distress. With the worldwide decline in pubertal onset age—particularly pronounced in East Asia—referrals for precocious puberty evaluation are rising, and timely diagnosis is essential to identify girls who will benefit from gonadotropin-releasing hormone (GnRH) analog therapy.

Diagnosis currently rests on the GnRH stimulation test, which confirms central HPG axis activation when peak luteinizing hormone (LH) exceeds a predefined threshold (commonly 5 IU/L) (1). However, the test requires serial blood sampling over 60–120 min, is invasive and distressing for young children, and demands considerable laboratory resources. Basal serum LH is a simpler candidate, but its performance is inconsistent across studies and assay platforms (2, 3) largely because pulsatile LH secretion renders a single measurement poorly reproducible.

Urinary LH offers a biologically appealing alternative: accumulation in the bladder integrates multiple secretory pulses, smoothing the sampling-time variability of serum measurements (4, 5), and first-morning voids additionally capture the sleep-entrained nocturnal LH surge of early puberty with acceptable day-to-day reproducibility (6–8). Urine collection is completely noninvasive and feasible at home. Studies in American, Korean, and European cohorts have reported encouraging diagnostic performance for urinary LH (AUC 0.84–0.88) (9–11), and Chinese data, though still limited, are promising (12–14). Nevertheless, most studies enrolled fewer than 200 participants, and reported cutoffs vary widely across assays and protocols; although recent reviews and a meta-analysis position urinary gonadotropins as complementary screening tools within tiered diagnostic algorithms (15, 16), no study has integrated urinary LH into a multivariable model that separates chronological age (CA) from skeletal maturation acceleration. Because bone age (BA) equals CA plus the BA − CA difference, these three quantities cannot be modeled together; entering CA and BA − CA as separate predictors disentangle a child’s position on the developmental timeline from the pathological skeletal acceleration characteristic of CPP.

We therefore conducted, to our knowledge, the largest single-center study of urinary LH in Chinese children, with three objectives: (i) to evaluate the diagnostic accuracy of first-morning urinary LH for CPP, including age-stratified performance and head-to-head comparison with basal serum LH; (ii) to establish sex- and pubertal stage–specific urinary LH reference intervals; and (iii) to develop two complementary prediction models based on the decomposition of bone age into CA and the BA − CA difference—a fully noninvasive model and a complete model incorporating serum gonadotropins—mirroring the stepwise clinical decision pathway.

## Materials and methods

This study is reported in accordance with the Standards for Reporting of Diagnostic Accuracy Studies (STARD) 2015 guidelines (17, 18).

### Study design and participants

This retrospective diagnostic accuracy study was conducted at Shenzhen Maternity and Child Healthcare Hospital, a tertiary pediatric endocrine referral center in Shenzhen, China. A total of 808 children were enrolled between January 2024 and June 2025, comprising three subgroups: (i) 295 girls presenting with breast development before 8 years of age, of whom 146 were diagnosed with CPP and 149 were classified as non-CPP (premature thelarche with a negative GnRH stimulation test); (ii) 271 healthy girls recruited from pediatric health examination clinics; and (iii) 242 healthy boys recruited from the same clinics. Enrollment was non-consecutive; participants were included according to the eligibility criteria when complete GnRH stimulation test results and first-morning urinary LH measurements were available.

Data stratification was performed a priori. Boys (n = 242) were enrolled solely for the establishment of male reference intervals and were excluded from all diagnostic analyses, as no male CPP cases were available in this cohort. Reference intervals were established exclusively in the healthy children (271 girls and 242 boys, n = 513); girls with early breast development were not included in reference interval estimation. Receiver operating characteristic (ROC) analysis of urinary LH was performed primarily in the symptomatic cohort (295 girls with early breast development: 146 CPP and 149 non-CPP) and additionally in an extended cohort of 566 girls that also included the 271 healthy controls, to characterize performance across the full screening spectrum. Multivariable logistic regression was performed in 295 girls (all CPP and non-CPP cases had complete data for all candidate predictors). The flow of participants through the study and the composition of each analysis set are summarized in **Figure 1**. Approximately 80 participants in the present study were also enrolled in an ongoing multicenter study led by our group, which has not yet been published; no participant in the present study has appeared in any previous publication. The objectives and analyses of the present study—urinary LH diagnostic accuracy, bone-age-decomposition prediction models, and reference intervals—are distinct from those of the multicenter study.

**Figure 1:**
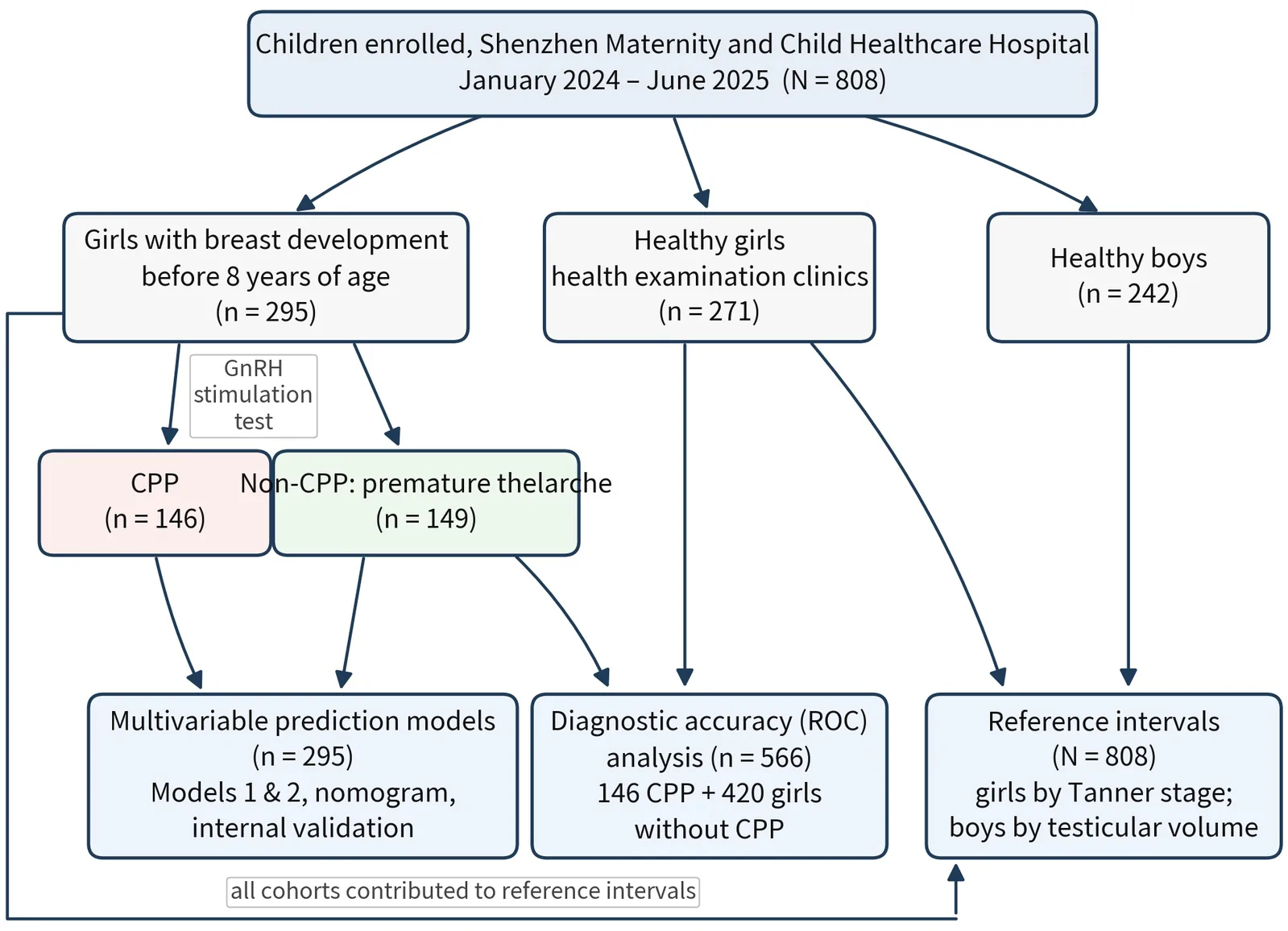
STARD flow diagram of participant enrollment and analysis sets. A total of 808 children were enrolled: 295 girls with breast development before 8 years of age (146 CPP, 149 non-CPP after GnRH stimulation testing), 271 healthy girls, and 242 healthy boys. Reference intervals were established in the 513 healthy children (271 girls and 242 boys); diagnostic accuracy (ROC) analysis was performed primarily in 295 symptomatic girls and additionally in an extended cohort of 566 girls including the healthy girls; multivariable prediction models were developed in 295 girls with complete data. CPP, central precocious puberty; GnRH, gonadotropin-releasing hormone; ROC, receiver operating characteristic; STARD, Standards for Reporting of Diagnostic Accuracy Studies.

The CPP group met the following criteria: (1) onset of breast development (Tanner stage B2 or greater) before 8 years of chronological age; (2) peak serum LH ≥ 5.0 IU/L after GnRH stimulation, with a peak LH/FSH ratio ≥ 0.6; (3) bone-age advancement assessed as supportive evidence (typically exceeding chronological age by more than 1 year), integrated with the stimulation-test result and the overall clinical picture; and (4) a first-morning voided urine specimen collected on the same day as, or within 72 h of, blood sampling. Bone-age advancement was not an absolute inclusion requirement: 25 of the 146 girls diagnosed with CPP (17.1%) had a BA − CA difference of ≤1 year at presentation, because the final diagnosis integrated the stimulation-test result with the overall clinical picture. Importantly, CPP classification was not dependent on the BA − CA difference: the diagnosis rested on the GnRH stimulation test result integrated with the overall clinical picture, with bone-age advancement used only as supportive evidence, and all 25 girls with a BA − CA difference of ≤1 year had unequivocally positive stimulation tests (peak LH ≥ 5.0 IU/L and peak LH/FSH ratio ≥ 0.6). The impact of the case definition on the prediction models was examined in a sensitivity analysis (see Results). The non-CPP group comprised girls with premature thelarche (breast development before 8 years) but a negative GnRH stimulation test (peak LH < 5.0 IU/L) and no evidence of pubertal progression during at least 6 months of follow-up. Exclusion criteria for both groups were: peripheral precocious puberty or organic causes (ovarian cysts, adrenal tumors, McCune–Albright syndrome, exogenous hormone exposure); prior GnRH analog treatment; incomplete GnRH stimulation test results; chronic systemic diseases; urinary tract infection or renal dysfunction; and missing urinary LH data.

Healthy controls were aged 3.0–12.0 years, had no history of endocrine disorders, showed normal physical development for age, had normal liver and renal function, and were taking no medications affecting hormonal status. Healthy girls were categorized by Tanner breast stage (B1–B4) assessed by experienced pediatric endocrinologists. Healthy boys were categorized by testicular volume measured with a Prader orchidometer: prepubertal (<4 mL), early pubertal (4–8 mL), and mid-to-late pubertal (>8 mL).

### Diagnostic criteria and bone age assessment

The reference standard for CPP diagnosis was the GnRH stimulation test. Triptorelin (0.1 mg) was administered subcutaneously, and blood samples were drawn at 0, 30, 60, and 90 min for LH and FSH measurement. CPP was confirmed when peak stimulated LH was ≥ 5.0 IU/L and the peak LH/FSH ratio was ≥ 0.6. Bone age was assessed by two independent pediatric radiologists using the Greulich–Pyle atlas method; the mean of the two readings was used, and a third radiologist adjudicated when the discrepancy exceeded 1 year.

### Laboratory assays

Morning fasting venous blood samples (8:00–10:00 AM) were collected for serum LH, FSH, estradiol (girls), and testosterone (boys) using a chemiluminescent immunometric assay (Beckman Coulter DxI 800; Beckman Coulter, Brea, CA, USA). Peak serum LH was defined as the maximum concentration across all post-stimulation time points.

First-morning voided urine specimens (5–10 mL) were collected on the morning of the clinic visit, stored at −20 °C, and analyzed in batches. Urinary LH, FSH, and creatinine were measured using a magnetic particle chemiluminescence immunoassay (Kangrun Biotechnology Co., Ltd., Guangzhou, China) on a fully automated analyzer. For urinary LH, the analytical detection limit was 0.398 mIU/mL; values below this threshold were assigned half the detection limit (0.199 mIU/mL) to permit logarithmic transformation. The LH-to-creatinine ratio was calculated as urinary LH (mIU/mL) divided by urinary creatinine (μmol/L), multiplied by 1,000. Within-run and between-run coefficients of variation were <5% and <8%, respectively. All assays were performed by the same laboratory technician following standardized operating procedures, with quality control at two concentration levels in each analytical batch. Laboratory personnel who performed the urinary and serum assays were blinded to the clinical diagnosis and group assignment, and the two pediatric radiologists who assessed bone age were blinded to the hormonal results and clinical classification.

### Statistical analysis

Analyses were performed using SPSS version 26.0 (IBM Corp., Armonk, NY, USA) and R version 4.3.0 (R Foundation for Statistical Computing, Vienna, Austria). All tests were two-tailed, and P < 0.05 was considered statistically significant unless otherwise specified.

#### Descriptive statistics and group comparisons

The distribution of continuous variables was assessed with the Shapiro–Wilk test. Normally distributed variables are reported as mean ± standard deviation (SD) and were compared between groups using independent-samples t-tests, with Cohen’s d as the effect size. Non-normally distributed variables are reported as median (interquartile range, IQR) and were compared using the Mann–Whitney U test, with Cliff’s delta as the effect size. Categorical variables were compared using the chi-square test or Fisher’s exact test, as appropriate.

#### ROC analysis and cutoff determination

ROC curves were constructed for urinary LH, the LH-to-creatinine ratio, and urinary FSH to evaluate diagnostic performance for CPP. The AUC was calculated with 95% confidence intervals (CI) using bootstrap resampling. Optimal cutoffs were determined by the Youden index (19). Sensitivity, specificity, positive predictive value (PPV), and negative predictive value (NPV) were calculated at each cutoff. Pairwise AUC comparisons were performed using the DeLong test for correlated ROC curves (20). Age-stratified ROC analyses were performed for the subgroups <7 years, 7–8 years, and >8 years, with bootstrap-based pairwise comparisons and Bonferroni correction. A head-to-head comparison of urinary LH versus basal serum LH was performed within the case cohort (n = 295) using the DeLong test. Because the clinically relevant question is whether urinary LH distinguishes CPP from non-progressive variants among girls presenting with early breast development, the symptomatic cohort (n = 295) was prespecified as the primary diagnostic analysis set; the extended cohort (n = 566), which additionally included healthy controls, was analyzed separately and is reported as a spectrum-extended estimate.

#### Multivariable logistic regression and bone age decomposition

Because BA, CA, and the BA − CA difference satisfy the deterministic identity BA = CA + (BA − CA), the three quantities cannot be entered simultaneously into a regression model without inducing perfect multicollinearity. We therefore entered CA and the BA − CA difference as two separate continuous predictors, allowing the model to distinguish (i) the age-related increase in the background probability of HPG axis activation from (ii) the pathophysiological acceleration of skeletal maturation. Clinically, a 6-year-old girl with a bone age of 8 years carries different diagnostic implications from an 8-year-old girl with a bone age of 10 years, although both exhibit a 2-year BA − CA difference.

Candidate predictors comprised CA, BA − CA difference, urinary LH, LH-to-creatinine ratio, urinary FSH, body mass index (BMI), breast Tanner stage, uterine length, basal serum LH, and basal serum FSH. All candidates were screened using univariable logistic regression, and variables with P < 0.10 were eligible for the multivariable model. Multicollinearity was assessed using the Spearman correlation matrix and the variance inflation factor (VIF). The interaction between CA and BA − CA difference was tested with a product term using the likelihood ratio test; it was not significant (P = 0.295) and was excluded.

Two models were constructed to reflect the staged clinical evaluation process:

- Model 1 (noninvasive model): CA + BA − CA difference + urinary LH + breast Tanner stage + uterine length, incorporating only information obtainable without venipuncture.
- Model 2 (complete model): Model 1 predictors + basal serum LH + basal serum FSH.

Each model was fitted using stepwise selection with the Akaike information criterion (AIC). Adjusted odds ratios (aOR) with 95% CI are reported for retained variables. Model discrimination was assessed with the C-statistic (equivalent to the AUC) with bootstrap 95% CIs, and the proportion of variance explained with Nagelkerke R². Calibration of Model 1 was evaluated with the Hosmer–Lemeshow test (21). Models were formally compared using the likelihood ratio test. Because stepwise selection retained slightly different predictor sets in the two models (uterine length was retained in Model 1 but not in the stepwise-selected Model 2), we additionally fitted a strictly nested extended model comprising the exact Model 1 predictor set plus basal serum LH and FSH and repeated the likelihood ratio test on these nested models. A nomogram based on Model 1 was developed as a visual prediction tool.

##### Internal validation and sensitivity analyses

To guard against overfitting, both models were internally validated with 500 bootstrap resamples, repeating the entire modeling process (univariable screening and stepwise selection) in each resample; optimism was estimated as the mean difference between the C-statistic in the bootstrap sample and that obtained when the bootstrap model was applied to the original dataset, and optimism-corrected C-statistics were derived. Because Model 2 showed complete separation, it was re-fitted using Firth’s penalized logistic regression, which yields finite and stable coefficient estimates under separation. The incremental value of urinary LH beyond serum gonadotropins was quantified with the integrated discrimination improvement (IDI) and the continuous (category-free) net reclassification improvement (NRI), comparing Model 2 with a base model excluding urinary LH; 95% CIs were obtained from 1,000 bootstrap resamples. As further sensitivity analyses, LASSO logistic regression with 10-fold cross-validation was applied to all candidate predictors, and Model 1 was re-fitted with BA − CA dichotomized at >1 and >2 years to compare continuous versus categorized parameterizations.

#### Reference interval establishment

Sex- and development-specific reference values for urinary LH were established using the percentile method following CLSI EP28-A3c guidelines (22). The P3, P10, P25, P50, P75, P90, and P97 percentiles were calculated. For girls, values were stratified by Tanner breast stage (B1–B4); for boys, by testicular volume category. Pubertal-onset cutoffs were derived by ROC analysis with the Youden index (girls: B1 vs B2 or greater; boys: <4 mL vs ≥4 mL). Subgroups smaller than the CLSI-recommended 120 individuals per partition are flagged as exploratory.

### Sample size and power

For the diagnostic accuracy analysis, assuming an expected AUC of 0.75 for urinary LH against a null hypothesis of 0.50, with α = 0.05 (two-tailed) and 80% power, a minimum of 82 CPP cases and 82 controls was required; the actual sample (146 CPP cases and 420 non-CPP controls) substantially exceeded this minimum. For reference intervals, CLSI EP28-A3c recommends at least 120 reference individuals per partition; the prepubertal girl (B1, n = 183) and prepubertal boy (<4 mL, n = 196) groups met this criterion, whereas smaller subgroups (B3 and B4 girls; >8 mL boys) are presented as exploratory.

## Results

### Baseline characteristics

A total of 295 girls presenting with breast development before 8 years of age were enrolled, of whom 146 were diagnosed with CPP and 149 were classified as non-CPP after a negative GnRH stimulation test. The clinical, anthropometric, and hormonal characteristics of both groups are presented in **Table 1**.

**Table 1:** Comparison of clinical and hormonal characteristics between girls with CPP and non-CPP controls.

Variable	CPP (n = 146)	Non-CPP (n = 149)	Test	Statistic	P-value	Effect size (95% CI)
Age (years)	7.81 ± 1.32	6.56 ± 1.26	t-test	t = 8.348	<0.001	0.972 (0.731–1.213)
Height (cm)	131.83 ± 9.58	121.65 ± 10.23	t-test	t = 8.815	<0.001	1.027 (0.784–1.269)
Weight (kg)	30.13 ± 8.92	23.65 ± 5.80	t-test	t = 7.404	<0.001	0.862 (0.624–1.101)
BMI (kg/m²)	17.03 ± 2.88	15.79 ± 2.10	t-test	t = 4.254	<0.001	0.495 (0.264–0.727)
Bone age (years)	9.85 ± 1.73	7.68 ± 1.82	t-test	t = 10.510	<0.001	1.224 (0.975–1.473)
BA–CA difference (years)	2.04 ± 1.12	1.12 ± 1.17	t-test	t = 6.900	<0.001	0.804 (0.566–1.041)
Urinary LH (mIU/mL), median (IQR)	**1.85 (0.73–4.75)**	**0.82 (0.64–1.13)**	Mann–Whitney U	U = 15087	**<0.001**	0.387 (0.312–0.463)
Urinary FSH (mIU/mL), median (IQR)	8.78 (4.99–18.30)	8.01 (4.86–12.94)	Mann–Whitney U	U = 12087.5	0.099	0.111 (0.044–0.178)
Basal LH (IU/L), median (IQR)	**0.80 (0.32–2.05)**	**0.14 (0.10–0.23)**	Mann–Whitney U	U = 18875.5	**<0.001**	0.735 (0.641–0.830)
Basal FSH (IU/L), median (IQR)	**3.52 (2.15–5.72)**	**1.98 (1.51–2.80)**	Mann–Whitney U	U = 15726.5	**<0.001**	0.446 (0.369–0.523)
LH/creatinine ratio, median (IQR)	**0.25 (0.10–0.52)**	**0.11 (0.08–0.17)**	Mann–Whitney U	U = 15055	**<0.001**	0.394 (0.318–0.469)
Uterine length (mm)	24.51 ± 5.83	20.54 ± 3.88	t-test	t = 6.905	<0.001	0.804 (0.567–1.041)

CA, chronological age; BA, bone age; IQR, interquartile range; BMI, body mass index. Normally distributed variables are presented as mean ± SD; non-normally distributed variables as median (IQR). Effect sizes are Cohen’s d for t-tests and Cliff’s delta for Mann–Whitney U tests; for reference, Cohen’s d ≥ 0.8 or Cliff’s delta ≥ 0.43 indicates a large effect, 0.5–0.8 or 0.28–0.43 a moderate effect, and smaller values a small effect. Significant between-group differences are highlighted in bold.

Girls with CPP were significantly older (7.81 ± 1.32 vs 6.56 ± 1.26 years; t = 8.348, P < 0.001, Cohen’s d = 0.972), taller (131.83 ± 9.58 vs 121.65 ± 10.23 cm; t = 8.815, P < 0.001, d = 1.027), and heavier (30.13 ± 8.92 vs 23.65 ± 5.80 kg; t = 7.404, P < 0.001, d = 0.862) than non-CPP controls, and had a higher BMI (17.03 ± 2.88 vs 15.79 ± 2.10 kg/m²; t = 4.254, P < 0.001, d = 0.495). Bone age was substantially advanced in girls with CPP (9.85 ± 1.73 vs 7.68 ± 1.82 years; t = 10.510, P < 0.001, d = 1.224), yielding a greater BA − CA difference (2.04 ± 1.12 vs 1.12 ± 1.17 years; t = 6.900, P < 0.001, d = 0.804). Uterine length was greater in the CPP group (24.51 ± 5.83 vs 20.54 ± 3.88 mm; t = 6.905, P < 0.001, d = 0.804).

Urinary LH, urinary FSH, basal serum LH, and basal serum FSH all departed significantly from normality (Shapiro–Wilk, all P < 0.001) and were analyzed using nonparametric methods. Urinary LH was higher in the CPP group (median 1.85 mIU/mL [IQR 0.73–4.75] vs 0.82 mIU/mL [IQR 0.64–1.13]; P < 0.001, Cliff’s delta = 0.387), as was the LH-to-creatinine ratio (median 0.25 [IQR 0.10–0.52] vs 0.11 [IQR 0.08–0.17]; P < 0.001, delta = 0.394). Basal serum LH (median 0.80 IU/L [IQR 0.32–2.05] vs 0.14 IU/L [IQR 0.10–0.23]; P < 0.001, delta = 0.735) and basal serum FSH (median 3.52 IU/L [IQR 2.15–5.72] vs 1.98 IU/L [IQR 1.51–2.80]; P < 0.001, delta = 0.446) were both elevated. Urinary FSH did not differ between groups (median 8.78 mIU/mL [IQR 4.99–18.30] vs 8.01 mIU/mL [IQR 4.86–12.94]; P = 0.099, delta = 0.111).

Bone age demonstrated the largest effect size (d = 1.224), followed by height (d = 1.027) and chronological age (d = 0.972), indicating that somatic maturation and age-related factors carry substantial discriminatory information. Basal serum LH showed a large nonparametric effect size (delta = 0.735), whereas urinary LH exhibited a moderate effect size (delta = 0.387), suggesting that urinary LH alone discriminates CPP less strongly than serum LH. The LH-to-creatinine ratio was approximately 2.3-fold higher in the CPP group (0.25 vs 0.11), indicating that creatinine correction amplifies between-group differences. No adverse events occurred in relation to first-morning urine collection or routine venipuncture.

### Diagnostic performance of urinary gonadotropins for CPP

In the primary analysis set—the symptomatic cohort of 295 girls presenting with early breast development (146 CPP vs 149 non-CPP)—urinary LH showed moderate diagnostic accuracy (AUC 0.692, 95% CI 0.627–0.752; cutoff 1.72 mIU/mL, sensitivity 51.4%, specificity 87.2%; **Table 2**). Within the symptomatic cohort, the LH-to-creatinine ratio achieved an AUC of 0.694 (95% CI 0.629–0.758; n = 294) and urinary FSH an AUC of 0.551 (95% CI 0.483–0.617); creatinine correction again conferred no significant improvement over uncorrected urinary LH (ΔAUC = 0.004, bootstrap P = 0.83). ROC analysis was additionally performed in the extended diagnostic cohort (n = 566; 146 CPP cases and 420 girls without CPP, including 271 healthy girls) to characterize performance across the full screening spectrum (**Table 3**, **Figure 2**).

**Table 2:** Head-to-head comparison of urinary LH versus basal serum LH for CPP diagnosis.

Marker	n	AUC (95% CI)	Cutoff	Sensitivity	Specificity
Urinary LH	295	0.692 (0.630–0.752)	1.72 mIU/mL	51.4%	87.2%
Basal serum LH	295	**0.868 (0.824–0.907)**	0.37 IU/L	**72.6%**	**90.6%**

Superior metrics are highlighted in bold.

**Table 3:** Diagnostic performance of urinary gonadotropins for CPP diagnosis.

Marker	n	AUC (95% CI)	Cutoff	Sensitivity	Specificity	PPV	NPV
Urinary LH	566	0.712 (0.663–0.762)	1.210 mIU/mL	60.3%	74.0%	44.7%	84.3%
LH/creatinine ratio	565	**0.717 (0.668–0.766)**	0.196	57.2%	**79.5%**	49.1%	84.3%
Urinary FSH	566	0.589 (0.533–0.646)	13.720 mIU/mL	34.2%	84.5%	43.5%	78.7%

AUC, area under the ROC curve; CI, confidence interval; PPV, positive predictive value; NPV, negative predictive value. Highest AUC and specificity are highlighted in bold.

**Figure 2:**
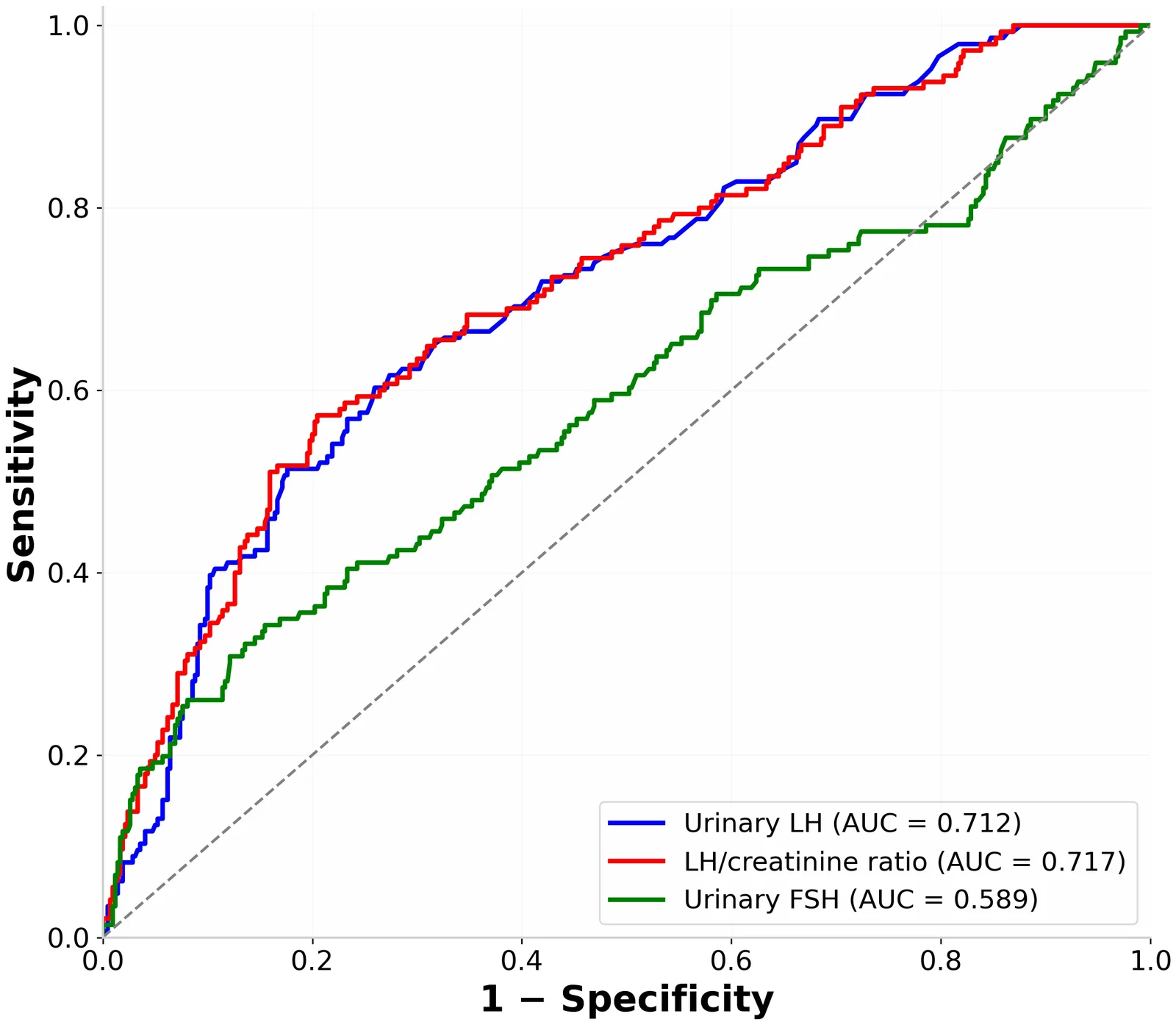
Receiver operating characteristic curves comparing urinary LH, the LH-to-creatinine ratio, and urinary FSH for CPP diagnosis (n = 566). AUC, area under the curve; LH, luteinizing hormone; FSH, follicle-stimulating hormone.

In the extended cohort, the LH-to-creatinine ratio achieved the highest AUC (0.717, 95% CI 0.668–0.766), followed closely by urinary LH (0.712, 95% CI 0.663–0.762). Urinary FSH showed substantially inferior discrimination (AUC 0.589, 95% CI 0.533–0.646), consistent with its negligible effect size in group comparisons. At the Youden-derived cutoffs, urinary LH yielded 60.3% sensitivity and 74.0% specificity, whereas the LH-to-creatinine ratio provided comparable sensitivity (57.2%) with higher specificity (79.5%). Both markers shared an NPV of 84.3% in the extended cohort; however, with sensitivities below 60% at the Youden-derived cutoffs, a negative result alone does not reliably exclude CPP and should be interpreted together with clinical follow-up. The PPV of urinary LH (44.4%) reflects the disease prevalence in the evaluated cohort.

Pairwise DeLong comparisons revealed no significant difference between the LH-to-creatinine ratio and urinary LH (Z = −0.61, P = 0.541). Urinary LH significantly outperformed urinary FSH (Z = 7.19, P < 0.001), as did the LH-to-creatinine ratio (Z = 5.67, P < 0.001). The marginal advantage of creatinine correction (ΔAUC = 0.005) suggests that either uncorrected urinary LH or the LH-to-creatinine ratio can be used for initial screening, with the latter offering modestly improved specificity.

### Age-stratified diagnostic accuracy

The diagnostic accuracy of urinary LH was evaluated across three age strata in the extended cohort (**Table 4**, **Figure 3**); these estimates include healthy girls and should be interpreted as spectrum-extended. Within the symptomatic cohort, the corresponding stratum-specific AUCs were 0.590 (95% CI 0.428–0.750) in girls <7 years (n = 106), 0.708 (95% CI 0.621–0.785) in girls aged 7–8 years (n = 151), and 0.500 (95% CI 0.255–0.751) in girls >8 years (n = 38, with only 6 non-CPP girls; this stratum estimate is markedly unstable), supporting the same conclusion that diagnostic utility peaks in the 7–8 years window. The age-stratified analyses in the extended cohort presented below are therefore regarded as secondary, spectrum-extended estimates.

**Table 4:** Age-stratified diagnostic performance of urinary LH for CPP diagnosis.

Age group	n	AUC (95% CI)	Cutoff (mIU/mL)	Sensitivity	Specificity
<7 years	164	0.679 (0.544–0.817)	1.50	50.0%	**90.7%**
7–8 years	161	**0.744 (0.652–0.828)**	1.21	61.2%	81.2%
>8 years	241	0.667 (0.590–0.733)	0.82	**79.5%**	48.8%

Highest AUC, specificity, and sensitivity are highlighted in bold.

**Figure 3:**
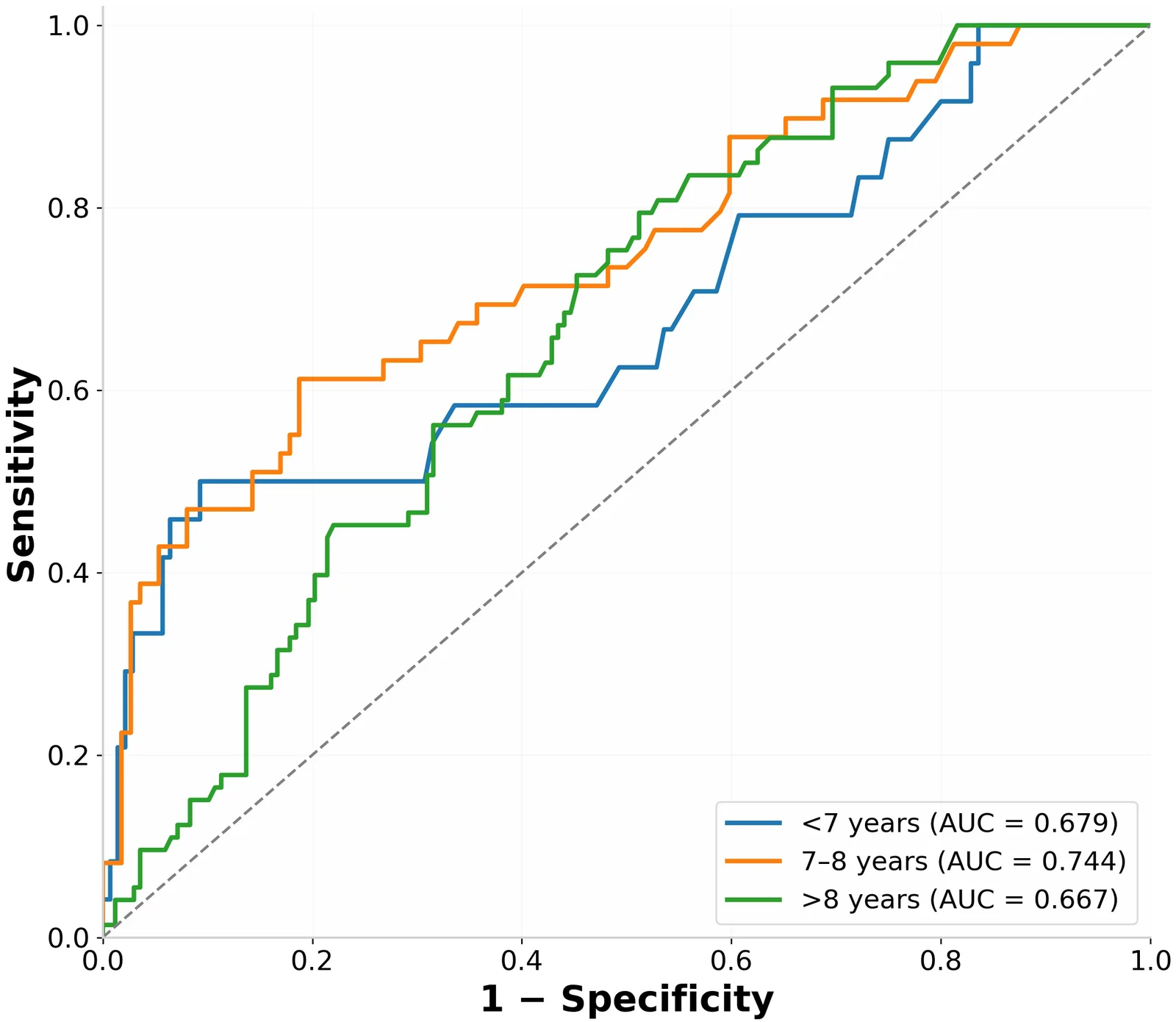
Age-stratified receiver operating characteristic curves for urinary LH in girls with suspected CPP. Solid line: <7 years (AUC = 0.679); dashed line: 7–8 years (AUC = 0.744); dotted line: >8 years (AUC = 0.667).

In the secondary, spectrum-extended analysis of the extended cohort, peak accuracy was likewise observed in the 7–8 years group (AUC 0.744, 95% CI 0.652–0.828; cutoff 1.21 mIU/mL; sensitivity 61.2%, specificity 81.2%), corresponding to the peak incidence window for CPP, in which the contrast between prepubertal quiescence and early HPG axis activation is most pronounced. In younger children (<7 years), the AUC was 0.679 (95% CI 0.544–0.817); at a cutoff of 1.50 mIU/mL, specificity was high (90.7%) but sensitivity low (50.0%), indicating that an elevated urinary LH in very young girls is highly specific for CPP but carries a substantial risk of false-negative results. In girls older than 8 years, the AUC declined to 0.667 (95% CI 0.590–0.733); at the lower cutoff of 0.82 mIU/mL, sensitivity rose to 79.5% but specificity fell to 48.8%, reflecting the increasing physiological overlap between normal early puberty and pathological precocity as girls approach the upper age limit for CPP diagnosis. Clinically, in girls younger than 7 years an elevated urinary LH (>1.50 mIU/mL) is highly specific for CPP (90.7%) and therefore has confirmatory value, whereas the low sensitivity (50.0%) means that a negative result cannot reliably exclude CPP; after 8 years, utility is limited by poor specificity. These extended-cohort, age-specific estimates and cutoffs are secondary analyses and require confirmation in symptomatic populations.

### Comparison between urinary LH and basal serum LH

A head-to-head comparison of urinary LH versus basal serum LH was conducted within the symptomatic cohort (the 295 girls with paired measurements), which constitutes the primary diagnostic analysis set (**Table 2**).

Basal serum LH demonstrated markedly superior diagnostic accuracy (AUC 0.868, 95% CI 0.824–0.907 vs 0.692, 95% CI 0.630–0.752), with both higher sensitivity (72.6% vs 51.4%) and specificity (90.6% vs 87.2%); the DeLong test confirmed that this difference was highly significant (Z = −6.23, P < 0.001; ΔAUC = 0.176). Basal serum LH therefore remains the superior single biomarker for CPP diagnosis. It should be emphasized that urinary LH (mIU/mL in urine, magnetic particle chemiluminescence assay) and basal serum LH (IU/L in serum, chemiluminescent immunometric assay) are measured in different biological matrices on different platforms; their absolute values and cutoffs are not directly comparable, and this comparison refers to diagnostic performance rather than concentrations. Nevertheless, the moderate performance of urinary LH, together with its adequate specificity (87.2%) and fully noninvasive collection, supports its use as a first-line screening tool rather than a replacement for serum gonadotropin assessment, particularly when integrated into multivariable models.

### Multivariable prediction models

#### Model development and variable selection

Two multivariable logistic regression models were constructed to mirror the clinical decision pathway from initial evaluation to definitive biochemical assessment. In univariable analyses, all ten candidate predictors were associated with CPP at P < 0.10: urinary LH (OR 1.572, 95% CI 1.329–1.860; C-statistic 0.692), LH-to-creatinine ratio (OR 22.020, 95% CI 6.275–77.275; C-statistic 0.694), chronological age (OR 2.174, 95% CI 1.740–2.718; C-statistic 0.764), BMI (OR 1.249, 95% CI 1.119–1.393; C-statistic 0.648), bone age (OR 1.988, 95% CI 1.674–2.360; C-statistic 0.803), BA − CA difference (OR 2.021, 95% CI 1.607–2.543; C-statistic 0.711), basal LH (OR 25.434, 95% CI 9.093–71.145; C-statistic 0.868), basal FSH (OR 1.623, 95% CI 1.394–1.889; C-statistic 0.723), breast Tanner stage (OR 5.879, 95% CI 3.401–10.163; C-statistic 0.699), and uterine length (OR 1.187, 95% CI 1.121–1.256; C-statistic 0.711).

Given the mathematical identity BA = CA + (BA − CA), chronological age and the BA − CA difference were entered as independent continuous variables, enabling the model to distinguish the effect of age itself from that of skeletal maturation acceleration. The interaction between CA and BA − CA difference was nonsignificant (likelihood ratio test, P = 0.295), supporting their inclusion as additive terms. Urinary LH and the LH-to-creatinine ratio were highly correlated (r = 0.823), so only urinary LH was retained; the correlation between CA and BA − CA difference was low (r = 0.215), the correlation between breast Tanner stage and uterine length—the two clinical examination variables—was modest (Spearman r = 0.320, P < 0.001), and all VIFs for retained variables were below 5 (maximum 2.14). Following stepwise selection with AIC optimization, two models were finalized (**Table 5**).

**Table 5:** Multivariable logistic regression prediction models for CPP diagnosis.

Variable	B	SE	Wald	P-value	Adjusted OR	95% CI
Model 1 (noninvasive, n = 295)						
Chronological age (years)	0.479	0.136	12.34	<0.001	1.614	1.236–2.108
BA–CA difference (years)	0.452	0.139	10.52	0.001	1.572	1.196–2.065
Urinary LH (mIU/mL)	0.305	0.089	11.85	<0.001	1.357	1.140–1.614
Breast Tanner stage	0.769	0.361	4.53	0.033	2.158	1.063–4.379
Uterine length (mm)	0.084	0.035	5.70	0.017	1.088	1.015–1.165
Constant	−8.317	—	—	<0.001	—	—
Model 2 (complete, n = 295)						
Basal LH (IU/L)	2.963	0.665	19.86	<0.001	19.357	5.258–71.261
Breast Tanner stage	0.670	0.398	2.83	0.092	1.954	0.896–4.261
Chronological age (years)	0.330	0.141	5.48	0.019	1.390	1.055–1.832
BA–CA difference (years)	0.388	0.149	6.81	0.009	1.474	1.101–1.972
Urinary LH (mIU/mL)	0.247	0.109	5.13	0.024	1.280	1.034–1.584
Basal FSH (IU/L)	−0.332	0.141	5.52	0.019	0.717	0.544–0.946
Constant	−5.327	—	—	<0.001	—	—

B, regression coefficient; SE, standard error; OR, odds ratio; CI, confidence interval; BA, bone age; CA, chronological age. Model 1 includes only noninvasive variables available at the initial clinical assessment; Model 2 adds serum gonadotropins. Both models were derived using stepwise selection with AIC optimization.

#### Model 1: noninvasive prediction model

Model 1 incorporated five predictors available at the initial clinical encounter without blood sampling: chronological age, BA − CA difference, urinary LH, breast Tanner stage, and uterine length. The model achieved a C-statistic of 0.853, with 73.3% sensitivity, 83.2% specificity, and an AIC of 294.8. The optimal cutoff probability derived by the Youden index was 0.504; as an internally derived value, this cutoff should not be regarded as a clinically established threshold and is reported to facilitate future external validation.

Both chronological age (aOR 1.614 per year, 95% CI 1.236–2.108, P < 0.001) and BA − CA difference (aOR 1.572 per year, 95% CI 1.196–2.065, P = 0.001) emerged as independent predictors, demonstrating that age itself and the degree of skeletal maturation acceleration each carry distinct diagnostic information. Urinary LH retained strong independent significance (aOR 1.357 per mIU/mL, 95% CI 1.140–1.614, P < 0.001), confirming its value as a noninvasive biomarker after adjustment for clinical and radiological parameters. Breast Tanner stage (aOR 2.158 per stage, 95% CI 1.063–4.379, P = 0.033) and uterine length (aOR 1.088 per mm, 95% CI 1.015–1.165, P = 0.017) also contributed independently.

Model calibration was good: the Hosmer–Lemeshow test yielded χ² = 9.73 (P = 0.284), and the Nagelkerke R² was 0.464. Model 1 thus provides well-calibrated prediction using entirely noninvasive data obtainable at the initial visit.

#### Model 2: complete prediction model

Model 2 added basal serum LH and FSH to the Model 1 variables. The C-statistic improved to 0.909 (cutoff probability 0.386), with 80.8% sensitivity, 83.2% specificity, and an AIC of 259.7; the Nagelkerke R² was 0.567.

Basal serum LH was the strongest independent predictor (aOR 19.357, 95% CI 5.258–71.261, P < 0.001), consistent with its established role as the biochemical cornerstone of CPP diagnosis. Chronological age (aOR 1.390, 95% CI 1.055–1.832, P = 0.019) and BA − CA difference (aOR 1.474, 95% CI 1.101–1.972, P = 0.009) retained independent significance after adjustment for serum gonadotropins. Urinary LH also maintained independent predictive value (aOR 1.280, 95% CI 1.034–1.584, P = 0.024), indicating that urinary LH provides complementary information beyond serum LH—likely reflecting the integrated secretion captured by urine accumulation versus the pulsatile variability of a single blood sample. This complementarity was formally supported by a significant integrated discrimination improvement (IDI 0.014, 95% CI 0.003–0.027), although the improvement in reclassification (continuous NRI 0.161) did not reach significance. The incremental value of urinary LH over serum gonadotropins is therefore real but modest, and urinary LH should be viewed as a complement to, rather than a replacement for, serum LH.

A notable finding was the significant inverse association of basal FSH (aOR 0.717, 95% CI 0.544–0.946, P = 0.019): after adjustment for basal LH and other covariates, higher FSH concentrations were associated with a lower probability of CPP. This finding is biologically interpretable—in true CPP, LH rises disproportionately relative to FSH, so that after controlling for LH, a higher residual FSH level favors nonprogressive premature thelarche over central activation.

Two aspects of the bone age decomposition warrant clarification. First, the nonsignificant CA × BA − CA interaction (P = 0.295) provides no evidence, in this sample, that the diagnostic weight of skeletal advancement differs by chronological age; however, statistical power to detect moderate interactions was limited, and this question merits testing in larger cohorts. Second, although clinicians often apply categorical rules such as BA − CA > 1 year, our sensitivity analysis showed that the continuous parameterization discriminated better (C-statistic 0.853 vs 0.844 for the >1-year and 0.841 for the >2-year dichotomization); the continuous form is therefore retained in the models, whereas the categorical rule remains a reasonable bedside approximation at a small cost of accuracy.

#### Model comparison and complete separation

The likelihood ratio test comparing Model 2 with Model 1 was highly significant (χ² = 37.11, P < 0.001), confirming the substantial incremental diagnostic value of serum gonadotropins and supporting a tiered workflow in which Model 1 is applied at initial presentation and Model 2 after serum results become available (**Figure 4**). The nomogram for Model 1 is presented in **Figure 5**. The strictly nested comparison—adding basal serum LH and FSH to the exact Model 1 predictor set—yielded a consistent result (likelihood ratio test χ² = 39.27, df = 2, P < 0.001; C-statistic 0.911), confirming that the incremental value of serum gonadotropins is not attributable to differences in predictor composition.

**Figure 4:**
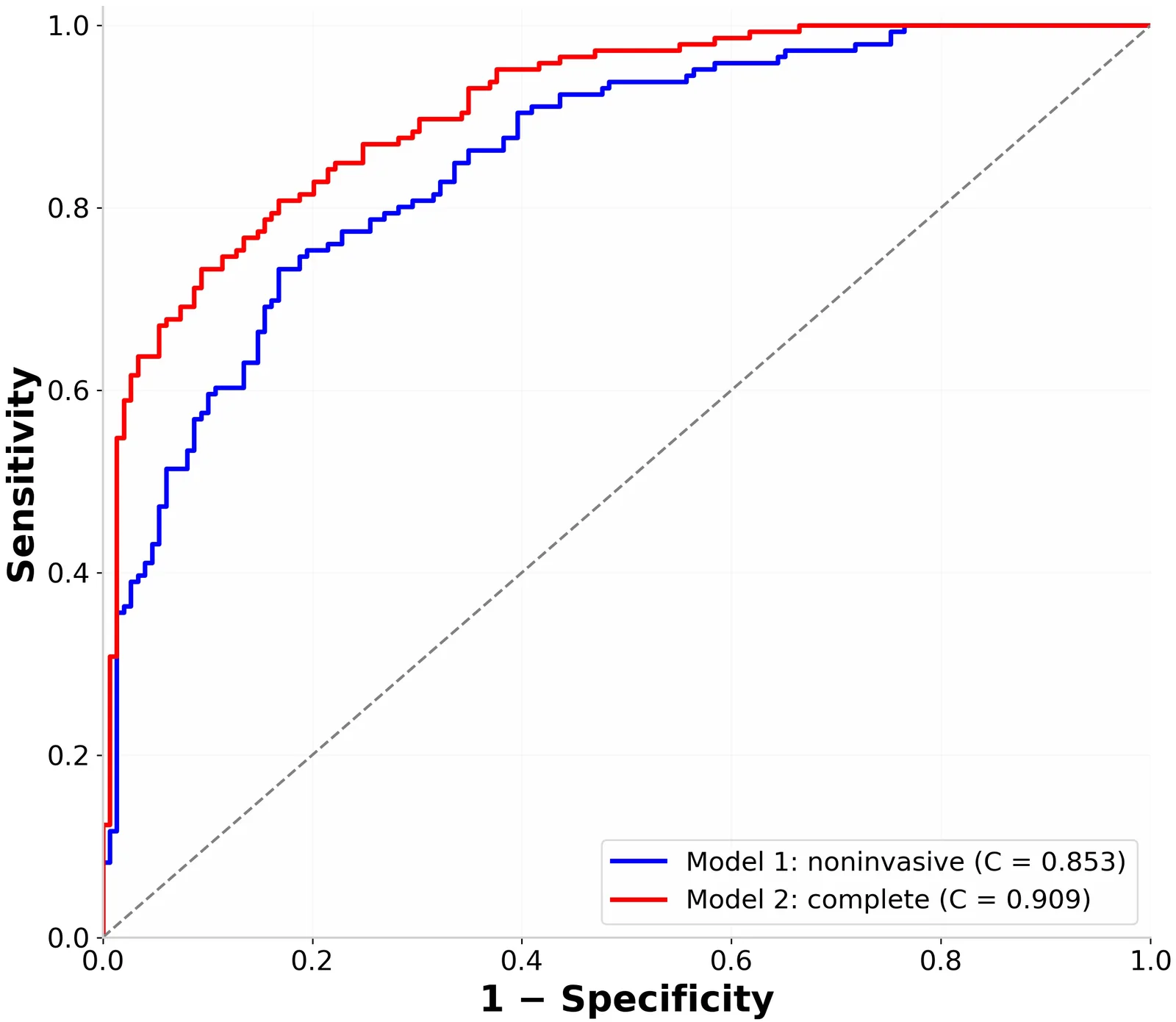
Receiver operating characteristic curves comparing Model 1 (noninvasive) and Model 2 (complete) for CPP diagnosis. Model 1 (blue line) achieves a C-statistic of 0.853 using only noninvasive variables; Model 2 (red line) achieves a C-statistic of 0.909 after adding serum gonadotropins.

**Figure 5:**
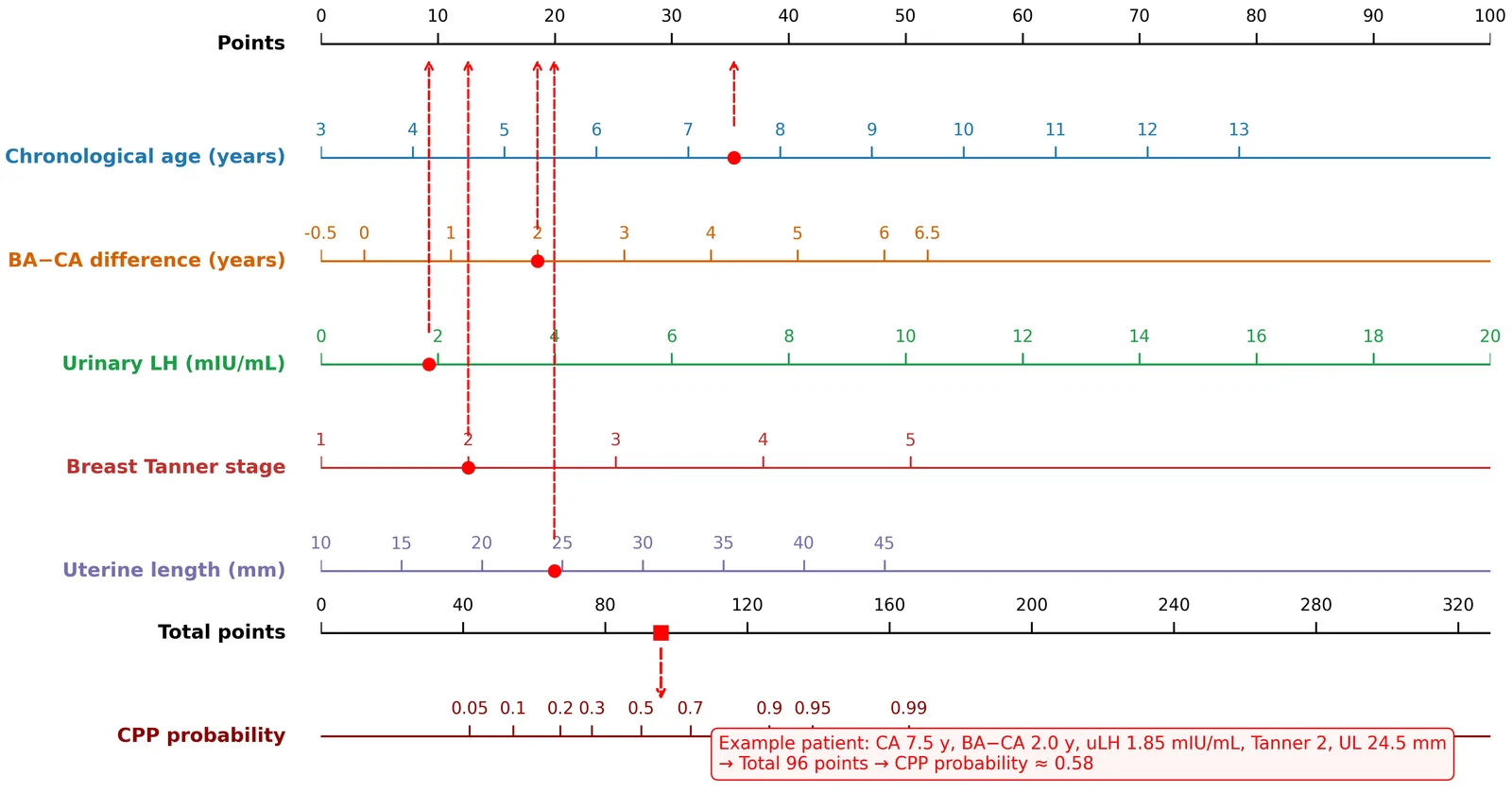
Nomogram for the noninvasive prediction model (Model 1), constructed from the regression coefficients in **Table 5**. To use the nomogram, locate the patient’s value on each predictor axis, read upward to the Points axis to obtain the score, sum the points across all five predictors, and project the total on the Total points axis downward to the CPP probability axis. The model cutoff probability of 0.504 corresponds to approximately 90 total points. The red dashed arrows illustrate scoring for an example patient (CA 7.5 years, BA–CA difference 2.0 years, urinary LH 1.85 mIU/mL, breast Tanner stage 2, uterine length 24.5 mm), who accumulates approximately 96 total points, corresponding to a CPP probability of approximately 0.58. Point values on each predictor axis are proportional to the product of that variable’s regression coefficient and its distance from the reference value; the predictor with the largest coefficient-weighted range defines the 100-point scale, and all other axes are projected proportionally. BA–CA, bone age minus chronological age difference.

Complete separation was observed in Model 2: 42 cases (14.2% of the analysis sample) exhibited predicted probabilities ≥ 0.99, all attributable to markedly elevated basal serum LH values. Complete separation can destabilize maximum-likelihood coefficient estimates; Model 2 was therefore re-fitted using Firth’s penalized logistic regression, and its calibration was evaluated for the penalized model (see Internal validation and sensitivity analyses). Discrimination metrics (C-statistic) and the likelihood ratio test remain valid under separation.

#### Internal validation and sensitivity analyses

Bootstrap internal validation (500 resamples, repeating the entire variable-screening and model-selection process in each resample) showed small optimism for both models (**Table 6**): the optimism-corrected C-statistic was 0.838 for Model 1 and 0.893 for Model 2, indicating that the reported discrimination is not substantially inflated by overfitting.

**Table 6:** Internal validation of the prediction models (500 bootstrap resamples).

Model	Apparent C-statistic	Mean optimism	Optimism-corrected C-statistic
Model 1 (noninvasive)	0.853	0.015	0.838
Model 2 (complete)	0.909	0.016	0.893

Re-fitting Model 2 with Firth’s penalized logistic regression yielded slightly shrunken but otherwise consistent coefficient estimates: basal LH remained the dominant predictor (penalized aOR 16.226, 95% CI 4.678–56.278, P < 0.001), and urinary LH (aOR 1.258, 95% CI 1.023–1.548, P = 0.030), BA − CA difference (aOR 1.448, P = 0.012), chronological age (aOR 1.371, P = 0.023), and basal FSH (aOR 0.732, P = 0.024) retained their directions and statistical significance, with a virtually unchanged C-statistic (0.908). Decile-based calibration of the penalized model showed close agreement between predicted and observed probabilities across most of the probability range (**Figure 6**): the observed CPP proportion tracked the mean predicted probability within each decile, with Wilson 95% confidence intervals overlapping the line of identity throughout; the largest deviations occurred in the mid-range deciles (predicted 0.26–0.45), where observed proportions modestly exceeded predictions. These results confirm that the coefficient estimates and inferences of Model 2 are stable despite complete separation.

**Figure 6:**
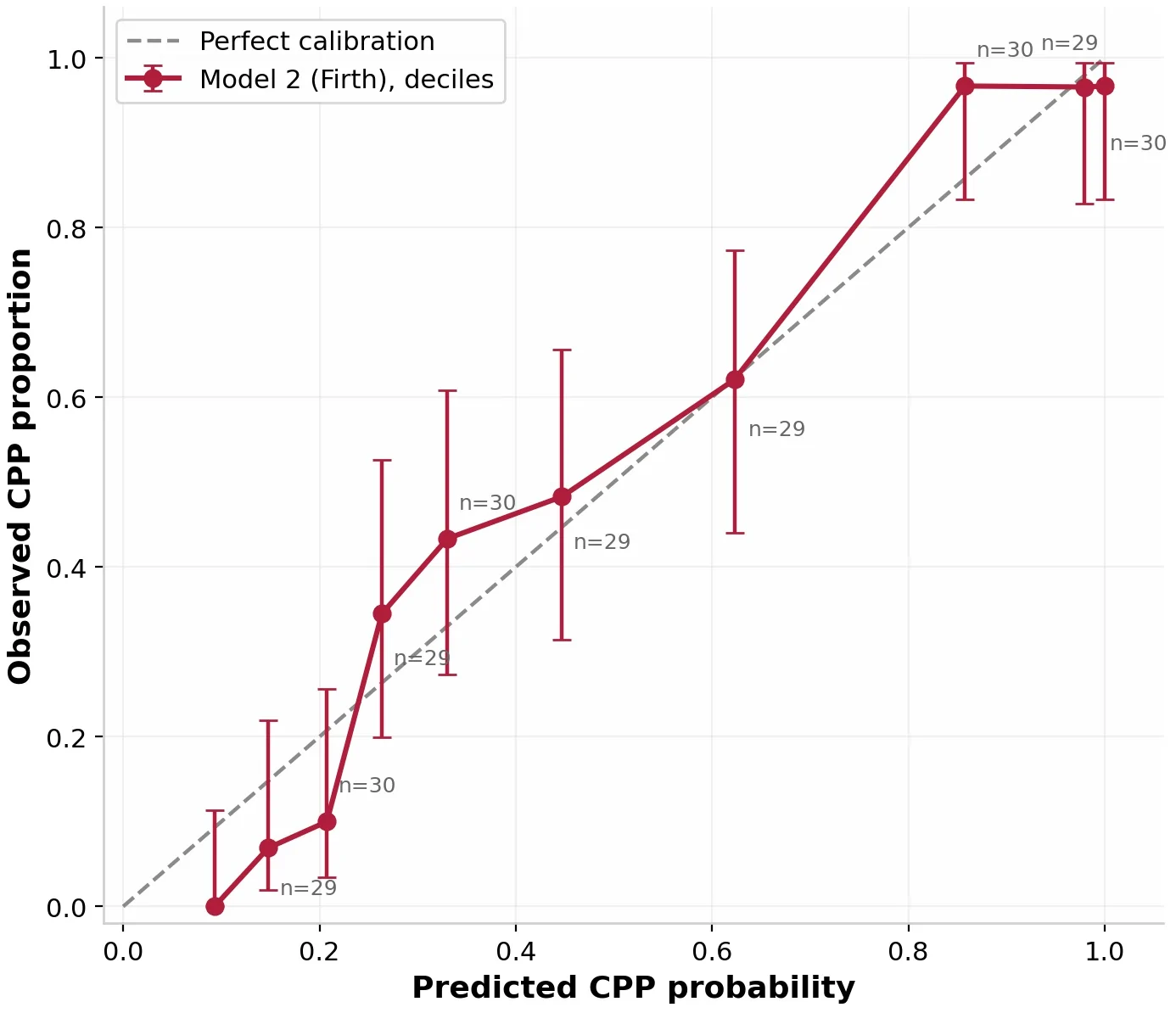
Calibration plot of the Firth-penalized Model 2 (n = 295). Predicted probabilities were grouped into deciles; each point shows the observed CPP proportion with 95% Wilson confidence intervals. The dashed diagonal line indicates perfect calibration. Group sizes are annotated.

The incremental value of urinary LH beyond serum gonadotropins was quantified by comparing Model 2 with a base model excluding urinary LH (C-statistic 0.900). Adding urinary LH improved the C-statistic to 0.909 (ΔAUC = 0.009) and yielded a significant integrated discrimination improvement (IDI = 0.014, 95% CI 0.003–0.027, P = 0.003); the continuous net reclassification improvement was positive but did not reach statistical significance (NRI = 0.161, 95% CI −0.064–0.505). Urinary LH thus provides a modest but statistically significant improvement in discrimination beyond basal serum gonadotropins.

LASSO logistic regression with 10-fold cross-validation, applied to all ten candidate predictors, retained chronological age, BA − CA difference, urinary LH, breast Tanner stage, uterine length, basal LH, and basal FSH with the largest penalized coefficients (C-statistic 0.915), corroborating the stepwise selection. Finally, re-fitting Model 1 with BA − CA dichotomized at >1 year (C-statistic 0.844) or >2 years (C-statistic 0.841, nonsignificant) yielded lower discrimination than the continuous parameterization (C-statistic 0.853), supporting retention of BA − CA as a continuous variable.

As a further sensitivity analysis addressing the CPP case definition, Models 1 and 2 were re-fitted after strictly enforcing the bone-age criterion (excluding the 25 girls with CPP who had a BA − CA difference of ≤1 year; n = 270: 121 CPP, 149 non-CPP); the C-statistics were 0.896 and 0.936, respectively (versus 0.853 and 0.909 in the primary analysis), and the direction and significance of the principal predictors (chronological age, BA − CA difference, and urinary LH) were unchanged, indicating that the findings are robust to the stringency of the CPP case definition.

### Reference intervals for urinary LH

#### Girls

Urinary LH concentrations across Tanner breast stages in 271 healthy girls are presented in **Table 7**. A progressive and significant increase was observed with advancing breast development (Kruskal–Wallis H = 60.078, P < 0.001). The median rose from 0.530 mIU/mL at B1 to 0.800 mIU/mL at B2 and then sharply to 6.450 mIU/mL at B3, reflecting the amplification of LH pulsatile secretion during mid-puberty. The 97th percentile increased from 2.625 mIU/mL at B1 to 9.250 mIU/mL at B2 and 13.595 mIU/mL at B3.

**Table 7:** Urinary LH reference values in girls by breast Tanner stage.

Breast stage	n	P50	P3	P25	P75	P97
B1	183	0.530	0.050	0.330	0.790	2.625
B2	60	0.800	0.178	0.520	2.050	9.250
B3	24	**6.450**	0.960	2.692	7.710	13.595
B4	4	1.855	0.955	1.357	3.298	6.195

Urinary LH in mIU/mL. Kruskal–Wallis H = 60.078, P < 0.001. The B2 (n = 60), B3 (n = 24), and B4 (n = 4) subgroups are below the CLSI EP28-A3c recommendation of 120 individuals per partition; values for these subgroups are exploratory.

Bold values indicate the median at the pubertal stage showing the sharp rise in urinary LH secretion.

The B4 subgroup comprised only four participants, and the B2 (n = 60) and B3 (n = 24) subgroups also fell below the CLSI-recommended 120 individuals per partition; reference values for these three subgroups should be considered exploratory and interpreted with caution. Using the B1 P97 (2.625 mIU/mL) as an upper reference bound and integrating ROC-derived thresholds, the urinary LH cutoff for pubertal onset in girls was determined as >1.02 mIU/mL (sensitivity 60.2%, specificity 83.1%). The considerable gap between the normative upper bound and the screening cutoff reflects the biological overlap between late prepubertal and early pubertal LH secretion; their distinct clinical uses are discussed under Clinical implications.

#### Boys

Urinary LH by testicular volume category in 242 healthy boys is presented in **Table 8**. A significant progressive increase was observed across categories (Kruskal–Wallis H = 30.197, P < 0.001), with medians of 0.530 mIU/mL in prepubertal boys (<4 mL), 0.990 mIU/mL in early puberty (4–8 mL), and 3.340 mIU/mL in mid-to-late puberty (>8 mL).

**Table 8:** Urinary LH reference values in boys by testicular volume.

Testicular volume	n	P50	P3	P25	P75	P97
<4 mL (prepubertal)	196	0.530	0.050	0.278	0.962	4.972
4–8 mL (early puberty)	32	0.990	0.197	0.595	2.040	7.523
>8 mL (mid-late puberty)	14	**3.340**	0.569	2.130	5.170	7.597

Urinary LH in mIU/mL. Kruskal–Wallis H = 30.197, P < 0.001. The 4–8 mL (n = 32) and >8 mL (n = 14) subgroups are below the CLSI EP28-A3c recommendation of 120 individuals per partition; values for these subgroups are exploratory.

Bold values indicate the median at the pubertal stage showing the sharp rise in urinary LH secretion.

The prepubertal median in boys (0.530 mIU/mL) was identical to that in girls at B1, suggesting comparable prepubertal urinary LH secretion between sexes. The pubertal-onset cutoff for boys was >0.95 mIU/mL (sensitivity 69.6%, specificity 74.0%). The 4–8 mL (n = 32) and >8 mL (n = 14) subgroups fell below the CLSI-recommended minimum of 120 individuals per partition, and their reference values should be considered exploratory.

Collectively, these data provide the first comprehensive sex- and pubertal stage–specific reference intervals for urinary LH in a Chinese pediatric population, established in healthy children (271 girls and 242 boys); because all pubertal subgroups (B2, B3, and B4 girls; 4–8 mL and >8 mL boys) were below the CLSI-recommended 120 individuals per partition, the corresponding estimates should be considered exploratory and preliminary pending larger studies. The pubertal-onset cutoffs (>1.02 mIU/mL in girls and >0.95 mIU/mL in boys) are remarkably concordant and approximate the 1.0 mIU/mL threshold proposed in European populations 8, supporting the biological validity of this threshold across ethnic groups.

## Discussion

### Principal findings

In this study of 808 Chinese children—to our knowledge the largest single-center evaluation of urinary LH for CPP to date—five principal findings emerge. First, first-morning urinary LH demonstrated moderate standalone diagnostic accuracy for CPP (AUC 0.692 in the symptomatic cohort; 0.712 in the extended cohort including healthy controls), with the LH-to-creatinine ratio performing marginally but not significantly better (AUC 0.717, DeLong P = 0.541), whereas urinary FSH was not discriminative. Second, diagnostic utility was age-dependent in the extended cohort, peaking in the 7–8 years window (AUC 0.744) and attenuating in younger (<7 years, AUC 0.679) and older (>8 years, AUC 0.667) girls. Third, decomposing bone age into CA and the BA − CA difference revealed that both variables carry independent predictive information, disentangling population-level age effects from individual-level HPG axis activation intensity. Fourth, the resulting two-model framework—a noninvasive Model 1 (C-statistic 0.853) and a complete Model 2 (C-statistic 0.909)—significantly improved diagnostic precision (likelihood ratio test χ² = 37.11, P < 0.001), with optimism-corrected C-statistics of 0.838 and 0.893 on bootstrap internal validation. Fifth, urinary LH retained independent predictive significance even after adjustment for basal serum LH (aOR 1.280, P = 0.024), indicating that urinary and serum LH provide complementary rather than redundant information. In addition, we established sex- and pubertal stage–specific urinary LH reference intervals in Chinese children, with pubertal-onset cutoffs close to 1.0 mIU/mL in both sexes. Because internal validation quantifies rather than eliminates optimism, real-world performance of the two models should be expected to lie closer to the optimism-corrected C-statistics (0.838 and 0.893) than to the apparent values (0.853 and 0.909), pending external validation.

### Comparison with previous studies

The moderate accuracy of urinary LH observed here is concordant with the pooled estimates of a recent meta-analysis of urinary gonadotropins for CPP diagnosis, which reported summary sensitivity and specificity of approximately 71% and 74% (16), strengthening confidence in the generalizability of moderate diagnostic performance across settings. The higher accuracy reported in some Korean studies (9, 10) may reflect differences in case spectrum, assay platforms, and collection protocols. In a prospective Danish cohort, first-morning-voided urinary total LH predicted central puberty onset with an AUC of 0.88 (8), exceeding our estimate; because both studies used first-morning specimens, this difference is more likely attributable to the prospective population-based design, the broader case spectrum, and differences in assay platforms and total-LH immunoreactivity measurement (7, 8). Zung et al. similarly reported that refined urinary LH thresholds improve the screening of progressive puberty in girls (23). The near-identical pubertal-onset cutoffs in girls (>1.02 mIU/mL) and boys (>0.95 mIU/mL) in our cohort are remarkably close to the 1.0 IU/L morning-urine threshold reported in the Danish cohort (8), lending cross-ethnic external validity to an approximately 1.0 mIU/mL threshold for pubertal transition. Our B1-stage P97 (2.625 mIU/mL) also aligns with the approximately 3.5 mIU/mL reported for Chinese prepubertal girls by Yao et al. (14), supporting the internal consistency of Chinese pediatric urinary LH distributions.

Our head-to-head comparison confirmed the superior standalone accuracy of basal serum LH (AUC 0.868 vs 0.692, P < 0.001), consistent with its established diagnostic role and with evidence that basal morning LH combined with clinical assessment can reduce unnecessary stimulation testing (2). Importantly, however, urinary LH retained independent significance in Model 2 after adjustment for serum LH. Several mechanisms may explain this complementarity: urinary LH integrates cumulative excretion over the bladder-filling interval and is therefore less vulnerable to the pulse-peak-or-nadir sampling artifact of a single blood draw (6, 7); it smooths the marked intra-individual variability of pulsatile secretion (7); and it may capture isoform or clearance patterns not fully reflected in circulating concentrations. The inverse association of basal FSH in Model 2 (aOR 0.717) is likewise biologically coherent: after controlling for LH, a higher residual FSH favors nonprogressive variants over central HPG axis reactivation, underscoring the diagnostic information carried by the LH-to-FSH relationship (5).

### Clinical implications

The two-model framework was designed to mirror the clinical decision pathway. Model 1 uses only variables obtainable at the first encounter—chronological age, BA − CA difference, urinary LH, breast Tanner stage, and uterine length—and achieved a C-statistic of 0.853 with 83.2% specificity, meaning that more than four in five girls without CPP could be correctly identified without venipuncture. A pragmatic tiered workflow follows, which should be regarded as preliminary because the models have undergone internal validation only: (i) at initial presentation, perform Tanner staging, bone age radiography, pelvic ultrasonography, and first-morning urine collection; (ii) apply Model 1 to estimate CPP probability; (iii) manage girls with low predicted probability (below the internally derived cutoff of 0.504, which is not a clinically established threshold) with observation; (iv) proceed to serum gonadotropin measurement in the remainder and apply Model 2; and (v) reserve GnRH stimulation testing for cases with intermediate or high Model 2 probability. The nomogram for Model 1 provides a bedside tool for rapid probability estimation. External validation in independent, preferably multicenter cohorts is required before this workflow and its cutoffs can be used in routine practice.

The clinical value of Model 1 extends beyond simply avoiding blood draws in a minority of patients. First, in primary care and community settings—where most girls with early breast development first present, and where pediatric endocrine assays and stimulation testing are unavailable—the noninvasive model provides an objective triage tool for rational referral, rather than referral of all cases or reliance on clinical impression alone. Second, its specificity and negative predictive value support a risk-stratification strategy: girls classified as low risk may be managed with structured clinical follow-up rather than immediate hormonal testing, sparing both venipuncture and the parental anxiety associated with testing. Because the sensitivity of urinary LH at the Youden-derived cutoffs is only approximately 60%, however, a low-risk classification or a value below the cutoff does not by itself exclude CPP, and repeat assessment is warranted whenever pubertal signs progress. Third, because first-morning urine can be collected at home and repeated without clinic visits, urinary LH–based assessment is uniquely suited to serial monitoring of girls with borderline findings who are managed expectantly. In resource-rich tertiary centers, Model 2 remains the preferred tool once blood results are available; the two-step framework thus allocates invasive testing to those most likely to benefit rather than competing with serum LH, consistent with reviews positioning urinary gonadotropins as complementary screening instruments within tiered algorithms 15,16. Pre-screening with this framework could also reduce the direct and indirect costs of unnecessary stimulation tests.

Two distinct urinary LH anchors derived in this study should not be used interchangeably. The pubertal-onset cutoff (>1.02 mIU/mL in girls), derived by ROC analysis contrasting prepubertal (B1) with pubertal (B2 or greater) healthy girls, should be distinguished from the CPP diagnostic cutoff (1.21 mIU/mL) derived in the diagnostic cohort; because both cutoffs were selected by the Youden index, which balances rather than maximizes sensitivity (sensitivity approximately 60% for each), neither value alone is sufficient to exclude pubertal activation or CPP. The prepubertal (B1) 97th percentile (2.625 mIU/mL) is a normative upper bound whose exceedance strongly suggests established HPG axis activation. We therefore propose a preliminary tiered interpretation for girls, to be confirmed by prospective external validation: <1.02 mIU/mL, lower likelihood—structured clinical follow-up with repeat urinary LH if signs progress, rather than reassurance alone; 1.02–2.6 mIU/mL, intermediate—proceed to the noninvasive model and serum gonadotropin testing; >2.6 mIU/mL, high suspicion—expedited pediatric endocrine evaluation. The wide gap between the screening cutoff and the normative bound illustrates the biological overlap between late prepubertal and early pubertal LH secretion, and reinforces that urinary LH is best used as a screening and triage tool rather than a stand-alone diagnostic test. The proposed tiered interpretation is summarized in **Table 9**.

**Table 9:** Proposed tiered interpretation of first-morning urinary LH in girls with early breast development.

Urinary LH (mIU/mL)	Interpretation	Suggested action
<1.02	Below the pubertal-onset screening cutoff; pubertal activation is less likely but not excluded (sensitivity approximately 60%)	Structured clinical follow-up; repeat urinary LH and reassessment if signs progress
1.02–2.6	Intermediate zone, between the pubertal-onset cutoff and the prepubertal upper reference bound (B1 P97 = 2.625)	Apply the noninvasive model (Model 1); proceed to basal serum gonadotropin measurement
>2.6	Exceeds the prepubertal 97th percentile; established HPG axis activation strongly suspected	Expedited pediatric endocrine evaluation, including serum gonadotropins and consideration of GnRH stimulation testing

Thresholds were derived in this cohort (screening cutoff by ROC analysis with the Youden index, which balances rather than maximizes sensitivity; upper bound by the percentile method per CLSI EP28-A3c) and should be considered preliminary, requiring prospective external validation before clinical implementation. HPG, hypothalamic–pituitary–gonadal; ROC, receiver operating characteristic.

Beyond diagnosis, urinary gonadotropins are increasingly used for treatment monitoring: urinary LH tracks the adequacy of GnRH analog suppression (24–26), and urinary FSH measured during triptorelin testing can monitor depot formulation efficacy (27, 28). Serial urinary LH measurements, interpreted against the prepubertal reference ranges established here, could eventually support ambulatory or home-based monitoring during the 1–3 years of typical GnRH analog therapy.

### Strengths and limitations

Strengths of this study include the large sample, which enabled robust age-stratified and decomposition analyses; the explicit sex stratification, with boys contributing exclusively to reference intervals; the two-model design that mirrors clinical decision-making; the first comprehensive male urinary LH reference data in a Chinese population; standardized first-morning void collection, which integrates overnight LH secretion and minimizes sampling-time variability (7, 8); standardized reporting following STARD 2015 (17, 18); and the methodological innovation of bone age decomposition, which is generalizable to other pediatric prediction settings.

Several limitations warrant consideration. First, the retrospective single-center design may have introduced variability in documentation and urine collection timing. Uterine length and Tanner staging were also assessed at a single center, and their reproducibility across operators, equipment, and institutions may be limited, potentially affecting model transportability. Second, all participants were of Chinese ethnicity, and multi-ethnic validation is required despite the cross-ethnic consistency suggested by the Danish data (8). Third, no boys with CPP were available, precluding diagnostic accuracy assessment in males, whose CPP is more frequently associated with organic central nervous system lesions (29). Fourth, although first-morning specimens were used to integrate overnight LH secretion, pre-analytical conditions—such as the interval between voiding and freezing, and hydration status—were not strictly standardized, which may have introduced variability in urinary LH concentrations (6, 7). Fifth, all samples were analyzed on a single assay platform; reported values are method-dependent, and cross-platform harmonization is needed before thresholds can be universally applied. Sixth, the GnRH stimulation test is itself an imperfect reference standard, and misclassification cannot be excluded. Seventh, advanced bone age is listed among the diagnostic criteria for CPP while the BA − CA difference also served as a model predictor, raising the possibility of incorporation bias; however, CPP classification was not dependent on the BA − CA difference—bone-age advancement served only as supportive evidence, all 25 girls with a BA − CA difference ≤1 year had positive GnRH stimulation tests, and the sensitivity analysis excluding these 25 girls yielded consistent—if anything stronger—model performance (C-statistics 0.896 and 0.936). Nevertheless, because the BA − CA difference is itself a model predictor, this sensitivity analysis cannot fully exclude incorporation bias, and the estimated effect of skeletal maturation should be interpreted with this caveat. Eighth, in the extended ROC analysis the disease-negative cohort combined girls with premature thelarche and healthy girls, which is expected to inflate apparent diagnostic performance relative to clinical practice; the symptomatic-cohort analysis (**Table 2**) therefore provides the more clinically relevant estimates, and the healthy-control-inclusive results should be interpreted as spectrum-extended. Ninth, the B4 girl (n = 4) and >8 mL boy (n = 14) subgroups fell below CLSI recommendations; their reference values are presented for exploratory purposes only and should not be used for clinical decision-making 22. Finally, Model 2 exhibited complete separation (42 cases, 14.2%, with predicted probabilities ≥ 0.99), reflecting the strong standalone discrimination of basal serum LH. This was addressed analytically with Firth’s penalized regression, which produced stable, finite coefficient estimates with unchanged conclusions (see Internal validation and sensitivity analyses); nevertheless, calibration of Model 2 could not be formally verified with the Hosmer–Lemeshow test, and predicted probabilities at the extreme range should be interpreted with caution.

### Future directions

We are planning a prospective multicenter external validation with predefined thresholds and standardized ultrasound training to assess inter-center reproducibility. Assay harmonization and home-based collection protocols are also needed. Future work should evaluate serial urinary LH for monitoring treatment response and explore its performance in boys with CPP and other pubertal disorders.

## Conclusion

First-morning urinary LH is a clinically viable noninvasive biomarker for CPP evaluation in girls, with its greatest utility realized within a structured multivariable framework rather than as a standalone test. Its diagnostic performance is moderate overall (AUC 0.692 in the symptomatic cohort; 0.712 in the extended cohort) and age-dependent, peaking in the 7–8 years window. Decomposing bone age into chronological age and the BA − CA difference showed that both carry independent predictive information; the resulting noninvasive Model 1 (C-statistic 0.853) enables risk stratification without blood sampling, and the complete Model 2 (C-statistic 0.909) refines risk once serum gonadotropins are available. The sex- and pubertal stage–specific reference intervals established here, with pubertal-onset cutoffs approximating 1.0 mIU/mL in both sexes, provide a framework for interpreting urinary LH in Chinese children. Internal validation indicated limited overfitting, and the coefficient estimates were stable under penalized regression; prospective external validation is the necessary next step. We suggest that first-morning urinary LH be incorporated as an initial triage component—rather than a stand-alone rule-out test—in girls presenting with early breast development, particularly those aged 7–8 years, with results interpreted within the two-model framework to guide selective use of serum gonadotropin measurement and GnRH stimulation testing; the proposed thresholds and tiered workflow remain preliminary until externally validated.

## Data Availability

The raw data supporting the conclusions of this article will be made available by the authors, without undue reservation.
